# Retinal single-layer analysis with optical coherence tomography (OCT) in schizophrenia spectrum disorder

**DOI:** 10.1192/j.eurpsy.2022.236

**Published:** 2022-09-01

**Authors:** T. Kregel, C. Schönfeldt-Lecuona, A. Schmidt, J. Kassubek, J. Dreyhaupt, R. Freudenmann, B. Connemann, M. Gahr, E. Pinkhardt

**Affiliations:** 1 Klinikum Christophsbad, Psychiatry And Psychotherapy, Göppingen, Germany; 2 University Clinic Ulm, Department Of Psychiatry And Psychotherapy Iii, Ulm, Germany; 3 University Clinic Ulm, Department Of Neurology, Ulm, Germany; 4 University Clinic Ulm, Institute Of Epidemiology And Medical Biometry, Ulm, Germany

**Keywords:** schizophrénia, Neuroimaging, optical coherence tomography, retina

## Abstract

**Introduction:**

Volume reductions in brain structures of patients with schizophrenia spectrum disorder (SSD) have repeatedly been found in voxel-based morphometry MRI studies. Hence, an underlying neurodegenerative etiological component of SSD is currently being discussed. In recent years, the imaging method of optical coherence tomography (OCT) has shown its potential in evaluating structural changes in the retina in patients with confirmed neurodegenerative disorders, providing a window into the brain.

**Objectives:**

To evaluate potential differences in measurements of retinal layers between patients with schizophrenia spectrum disorder and healthy controls with OCT.

**Methods:**

Twenty-six patients with schizophrenia or schizoaffective disorder and 23 age- and sex-matched healthy controls were examined with the Heidelberg Spectralis OCT system to derive a single-layer analysis of both retinas. The segmentation of retinal layers was manually corrected to minimize artifacts and software imprecisions.

**Results:**

Compared to the control group, SSD patients showed reduced thickness and volume measurements for nearly all retinal layers, and these differences reached significance for macular volume, macular thickness, retinal nerve fiber layer (RNFL) and inner nucleiform layer (INL). Furthermore, a significant correlation between the duration of illness and the total volume of the RNFL was found.

**Conclusions:**

Our OCT measurements demonstrate reduced single retinal layer thickness in patients with SSD. In the context of the MRI volume changes, our results provide further evidence that structural changes seen in the brain of patients are also observable in the retina, potentially allowing further insights into the different components of the nervous system that are altered in this highly etiologically complex disorder.

**Disclosure:**

No significant relationships.

